# Supplementary material for: Modelling the role of groundwater hydro-refugia in East African hominin evolution and dispersal
Source: Nat Commun. 2017 May 30;8:15696. doi: 10.1038/ncomms15696 (PMC5460002; doi:10.1038/ncomms15696)
Supplement: Supplementary Information — Supplementary Figures and Supplementary Tables [file ncomms15696-s1.pdf]

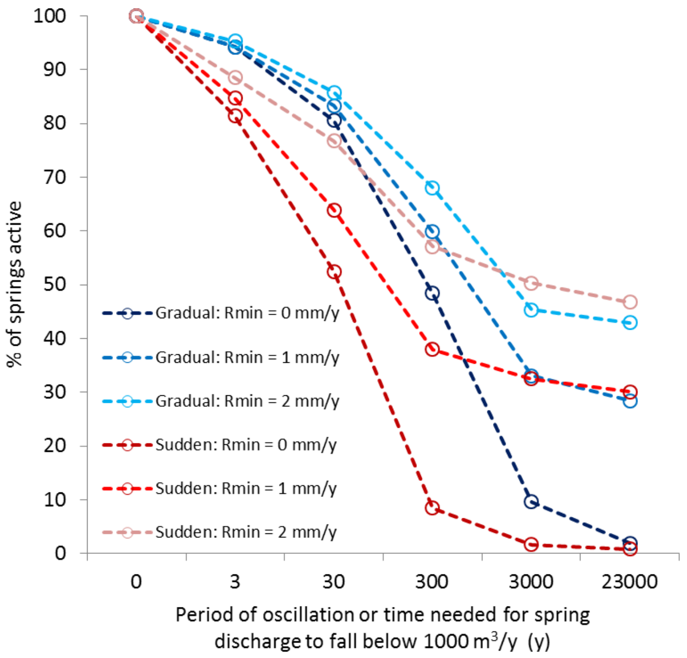


**Supplementary Figure 1**: Frequency of springs still active (i.e. flow rate still above 1000 $m^3$/y) at the driest part of climate cycles under gradual (periodic) and sudden (step) changes in recharge from modern day values to various minimum values (Rmin). All figures and calculations in the paper use the results for Rmin = 1 mm/y unless stated. While this is a conservative value for an arid climate, the Rmin = 0 end member provides a useful indication of the decline in active springs during any prolonged periods of zero rainfall (and therefore zero recharge).

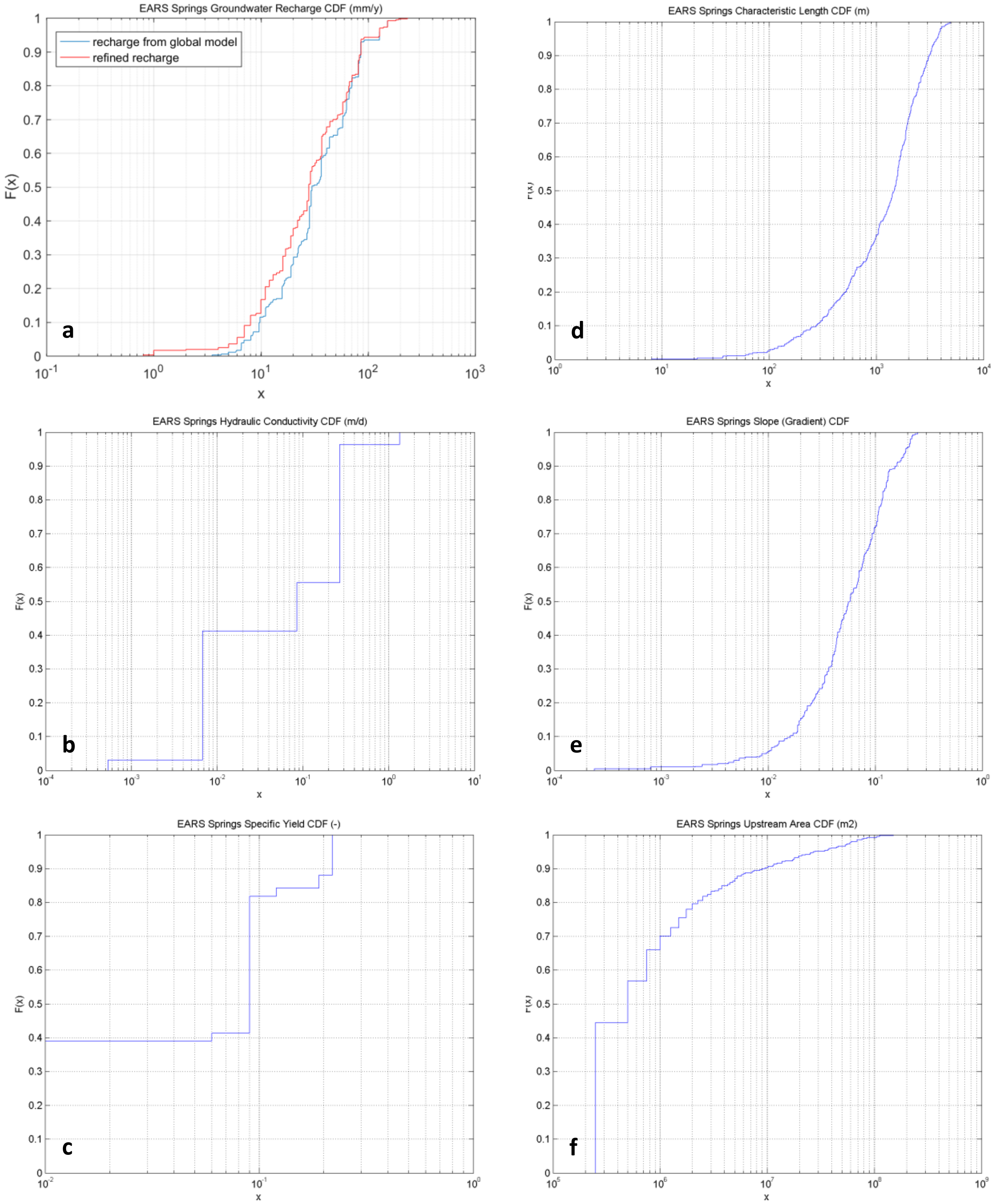


**Supplementary Figure 2:** Cumulative probability distributions (F(x)) for all mapped spring characteristic input parameters (x) used for modelling. **a**. groundwater recharge. **b**. hydraulic conductivity. **c**. specific yield. **d**. characteristic length. **e**. slope. **f.** catchment area.

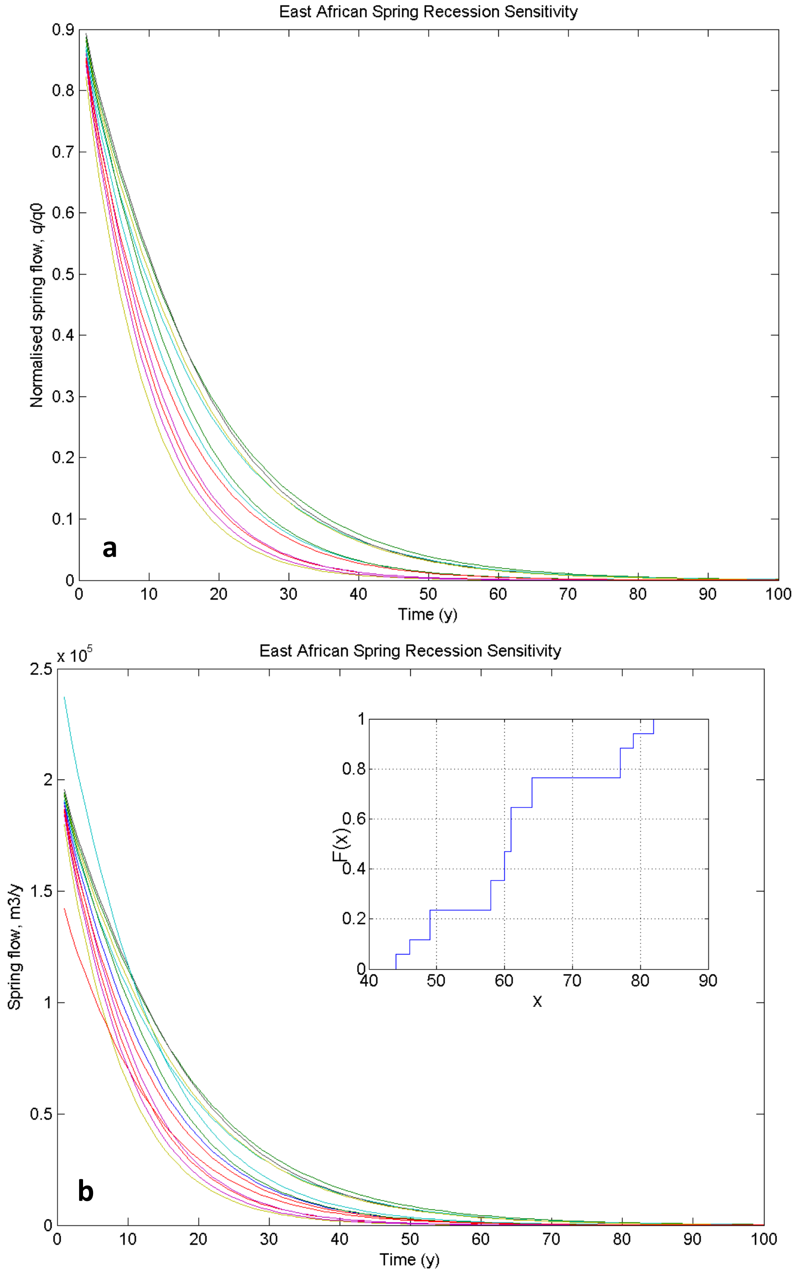


**Supplementary Figure 3:** Sensitivity of spring discharge, to a +/-25% variation from a baseline defined by an average of all parameters, to the sudden cessation of recharge from steady state conditions for **a.** normalised spring flow (q/q0) and **b**. absolute spring flow. The CDF (F(x)) for x = time in years to reach 1000 $m^3/y$ is shown as an inset. At the 95% confidence interval (i.e. 2 standard deviations), the range in x is +/-37%.

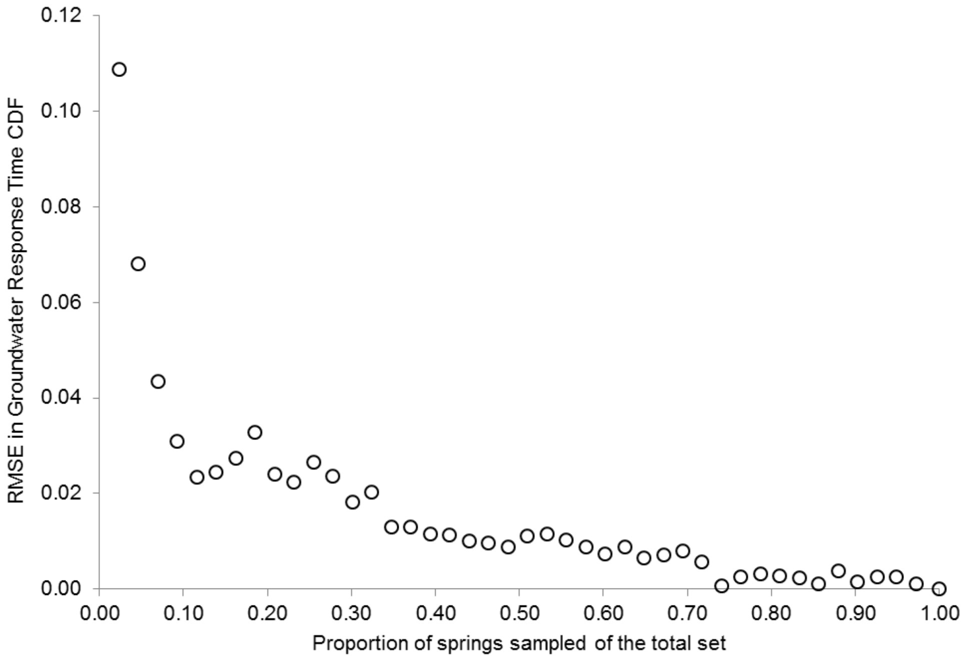


**Supplementary Figure 4:** Results of the statistical test for representativeness of spring sampling. Root mean squared error (RMSE) in the cumulative distribution function (CDF) of the groundwater response times plotted against increasing size of random subsamples as a proportion of the total mapped spring population.

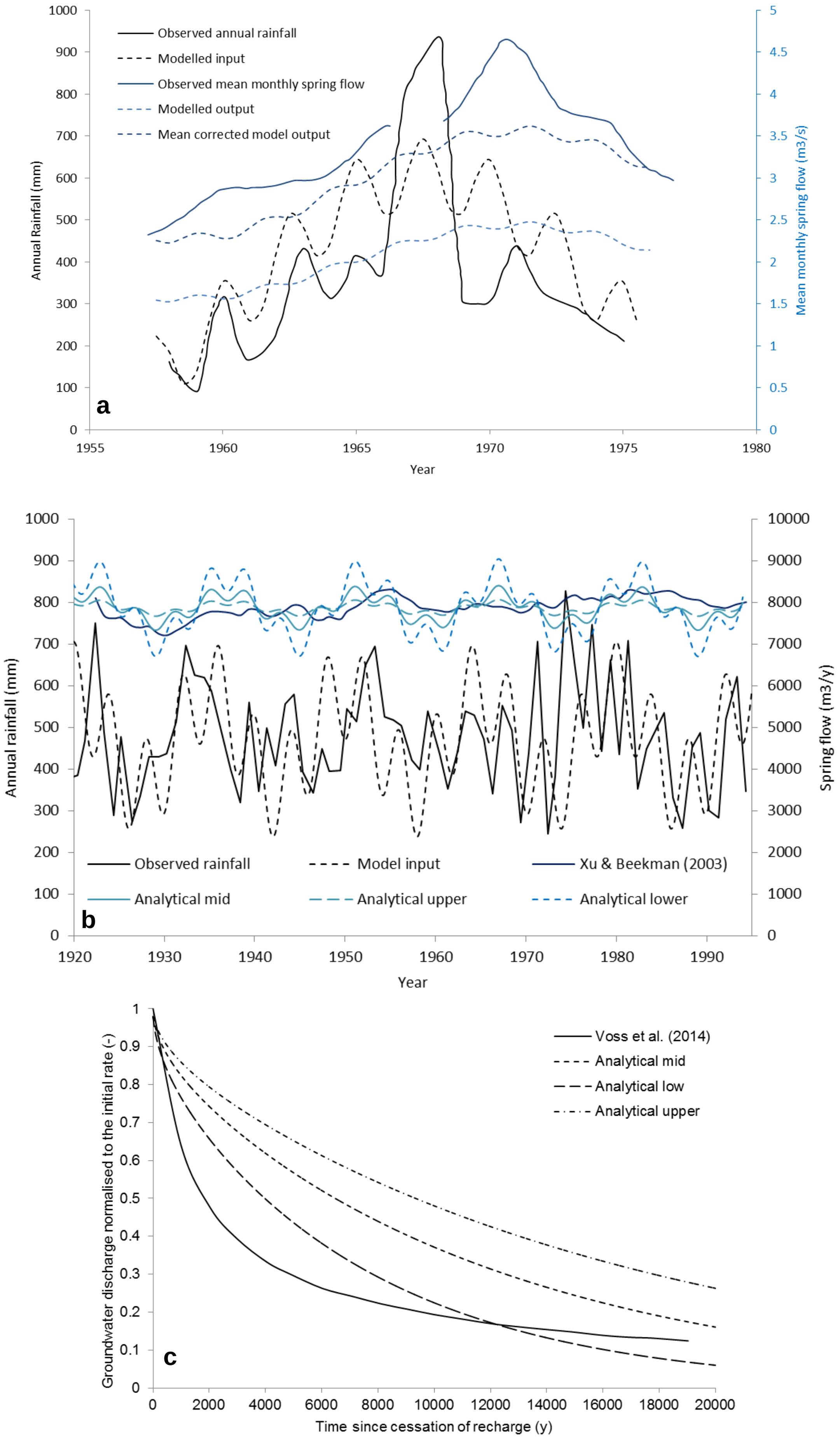
Observed annual rainfall
Modelled input
Observed mean monthly spring flow
Modelled output
Mean corrected model output
Annual Rainfall (mm)
Mean monthly spring flow (m3/s)
Year
a
Observed rainfall
Model input
Xu & Beekman (2003)
Analytical mid
Analytical upper
Analytical lower
Annual rainfall (mm)
Spring flow (m3/y)
Year
b
Voss et al. (2014)
Analytical mid
Analytical low
Analytical upper
Groundwater discharge normalised to the initial rate (-)
Time since cessation of recharge (y)
c

**Supplementary Figure 5:** Results of analytical model testing compared against: **a**. observed flows for Mzima Springs, Tanzania[54]. 'Modelled input' used signals with periods of 2.5 and 25 years with a ratio of amplitudes of 1:3, and hydraulic parameters uncalibrated from GIS mapping (see Methods) yielding a groundwater response time (GRT) of 37.5 y. 'Mean corrected model output' is a shifted plot of the raw model output to enable a better comparison to be made in the observed and modelled amplitude and attenuation despite the difference in observed and modelled absolute spring flow. **b**. naturalised flows for Uitenhage Spring, South Africa[55]. 'Modelled input' used signals with periods of 4 and 15 years with a ratio of amplitudes of 1:1 and the published range of hydraulic parameters yielding a GRT range of 17 y, 74 y and 530 y for 'Analytical low', 'mid' and 'upper' respectively. **c**. modelled flows for North African Nubian Aquifer System[56]. The published input parameters from the model were used as input parameters with estimates of average flowpath lengths of 300 km, 400 km and 500 km yielding GRT ranges of 25 ky, 44 ky and 68 ky for 'Analytical low', 'mid' and 'upper' respectively.

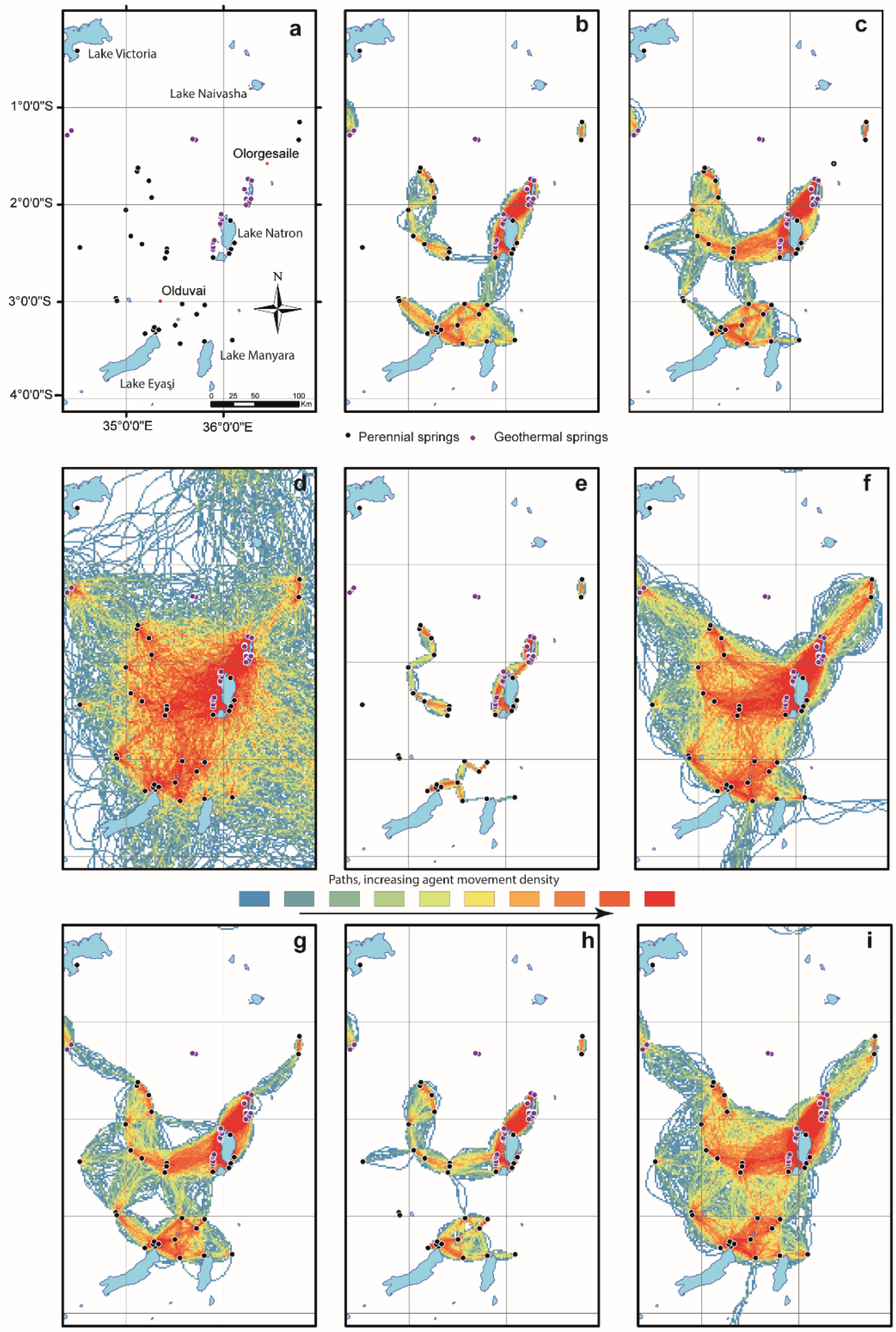


**Supplementary Figure 6:** Sensitivity of spring networks (hydro-refugia) to various cost maps and cost scaling in southern Kenya and northern Tanzania to demonstrate that spring networks exist in some form across all modelling scenarios. The scenarios shown are based on the springs persistent at 23 ky as the only active 'water patch'. **a**. Location details. **b**. Scenario using slope [Cost-1] as the cost layer. **c**. Scenario using roughness [Cost-2] as the cost layer. **d**. Scenario using no cost layer [Cost-0]. **e**. Scenario using roughness [Cost-2] with increased scaling [Scaling-2]. **f**. Scenario using roughness [Cost-2] with decreased scaling [Scaling-3]. **g**. Scenario using roughness [Cost-2] and a total three day travel distance of 180 km. **h**. Scenario using roughness [Cost-2] and a total three day travel distance of 120 km. **i**. Scenario using slope + roughness as the cost layer [Cost-5]. Unless otherwise stated all three day travel distances are 150 km and the turn angle is 20 degrees.

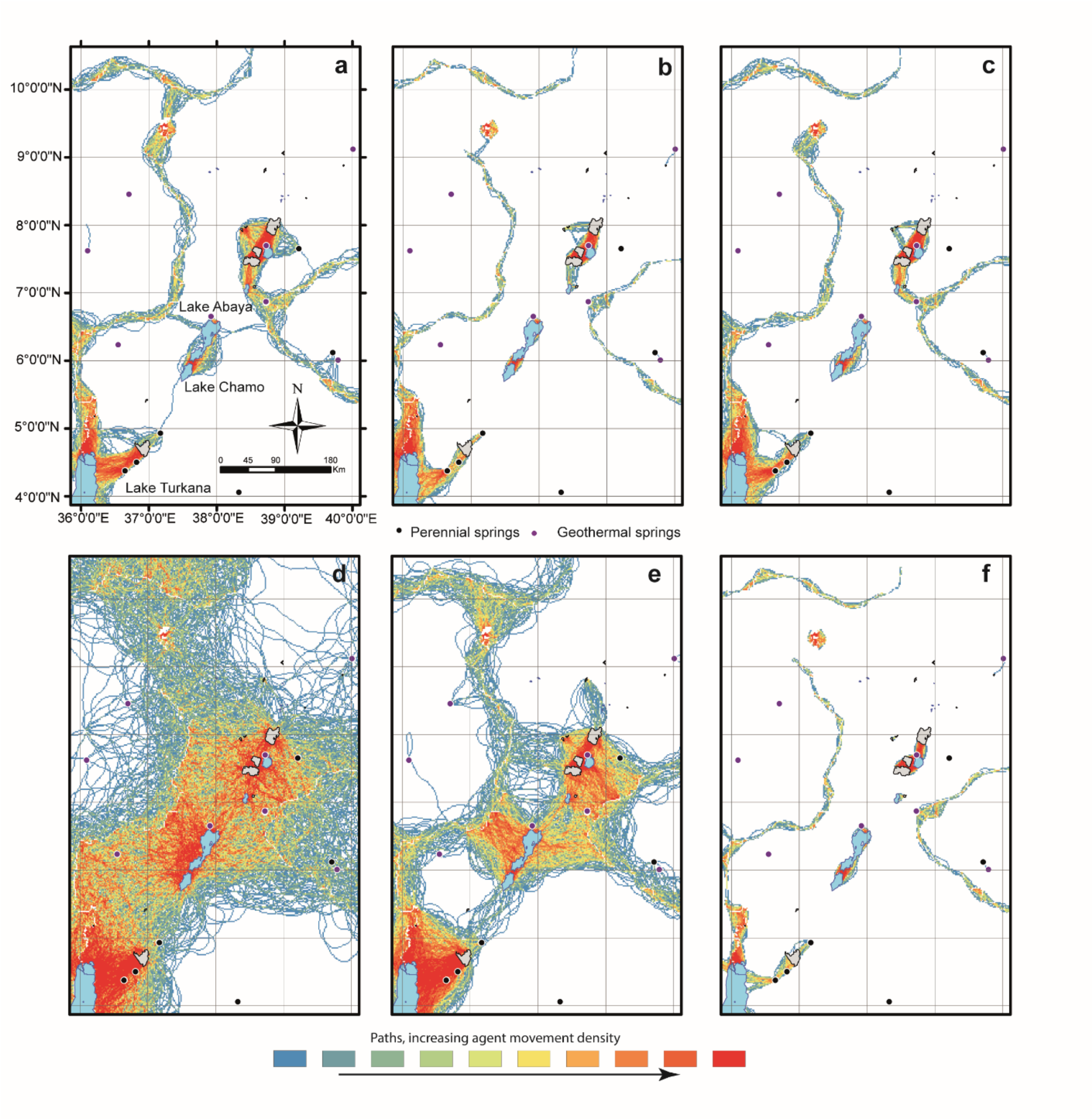


**Supplementary Figure 7:** Sensitivity of dispersal routes to various cost maps and cost scaling in southern Ethiopia and northern Kenya. The scenarios shown are based on the present day springs, lakes (fresh and saline), perennial wetlands, major rivers with flow >0 $km^3$/y. Three-day travel distance is set at 150 km unless otherwise stated. Note that the potential for east-west dispersal routes is sensitive to the model parameters used. **a**. Location details, plus paths using roughness [Cost-2] as the cost layer with the standard scaling [Scaling-1]. **b**. Same scenario but using slope [Cost-1] as the cost layer with the standard scaling [Scalling-1]. **c**. Same scenario but using slope [Cost-5] as the cost layer with the standard scaling [Scalling-1]. **d**. Same scenario but using no [Cost-0] cost layer. e. Location details, plus paths using roughness [Cost-2] as the cost layer with the standard scaling [Scaling-3]. **f**. Location details, plus paths using roughness [Cost-2] as the cost layer with the standard scaling [Scaling-3].

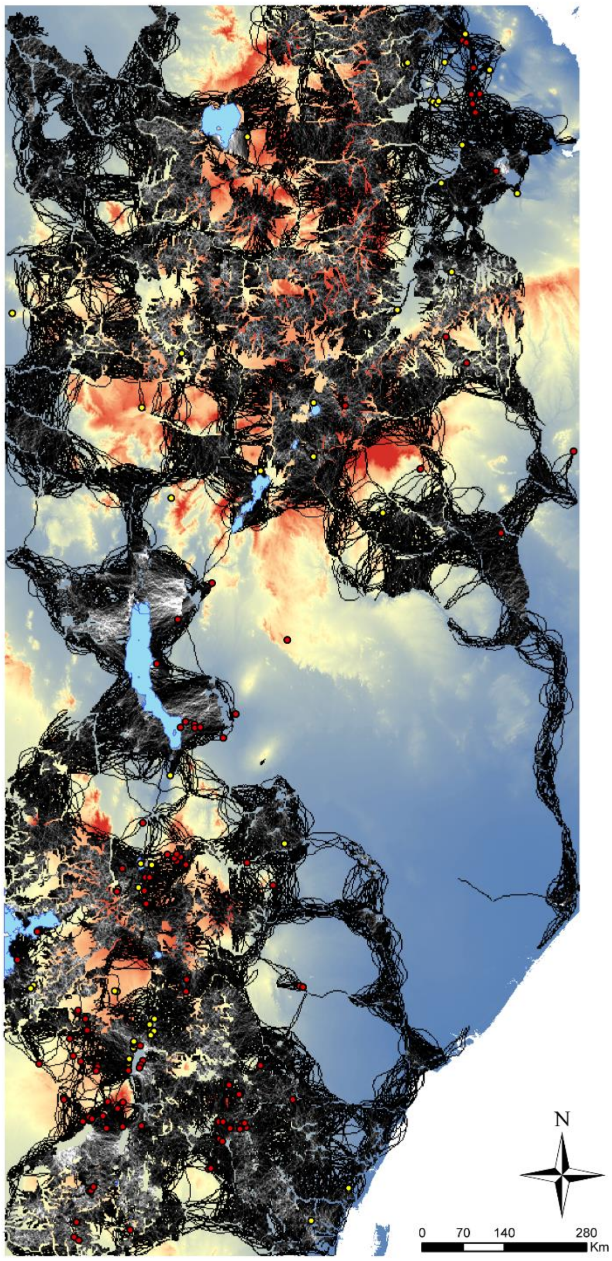


**Supplementary Figure 8:** Fourth modelled scenario based on the present day wet scenario (Run-2). Agent based modelling results based on a dry hydrological scenario using a maximum three-day travel distance of 150 km and surface roughness as the cost layer scaled according to Supplementary Figure 11. This scenario uses modern springs (seasonal, perennial + geothermal), wetlands (perennial), lakes (fresh + saline), major rivers with a flow >0 $km^3$/y, and perennial streams mapped from the 1:500,000 scale maps. The black lines shown represent the tracks of agents in the model. Note the potential for widespread dispersal.

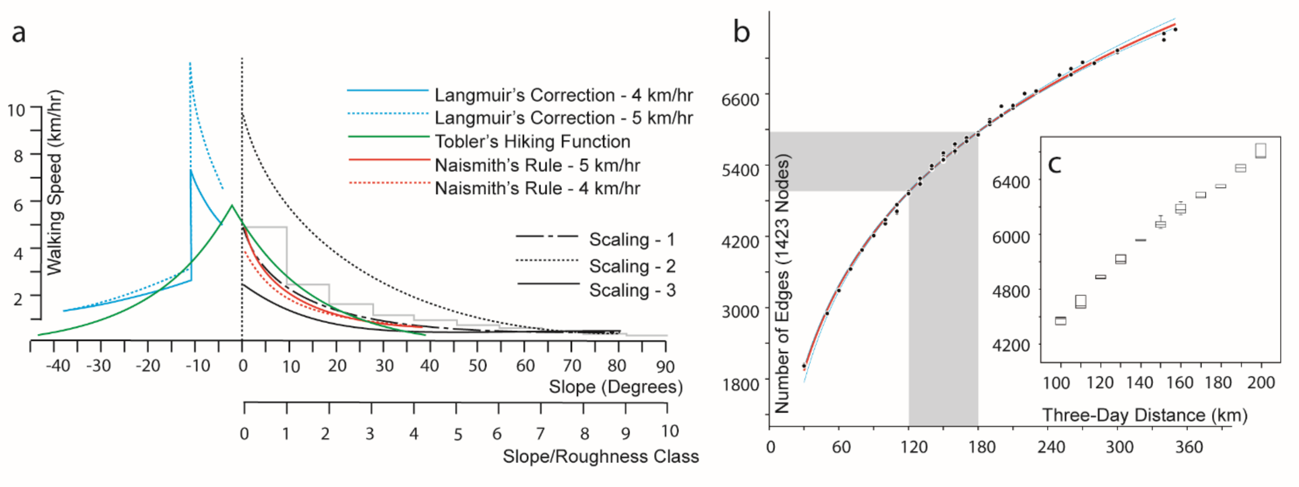


**Supplementary Figure 9: Cost calibrations for slope and roughness and model sensitivity. a**. Shows the impact on walking speed of slope based on Naismith's Rule as expressed by Rees *et al.* (2004)[61]. Three possible scaling scenarios are also shown. Scaling-2 was used for both slope and roughness as the default modelling option. The grey stepped line shows its transformation to modelling data classes. **b**. Variation of network density with travel distance and comparison of the modelled scenarios. a. shows the relationship between three day travel distance and the number of edges within a network (Run-3). The data follows a power function, with 95% confidence limits shown (N=63). **c.** Inter-run variation at a given distance (N=5). Note the inter-run variation is less than the variation between each increase in distance step (10 km).

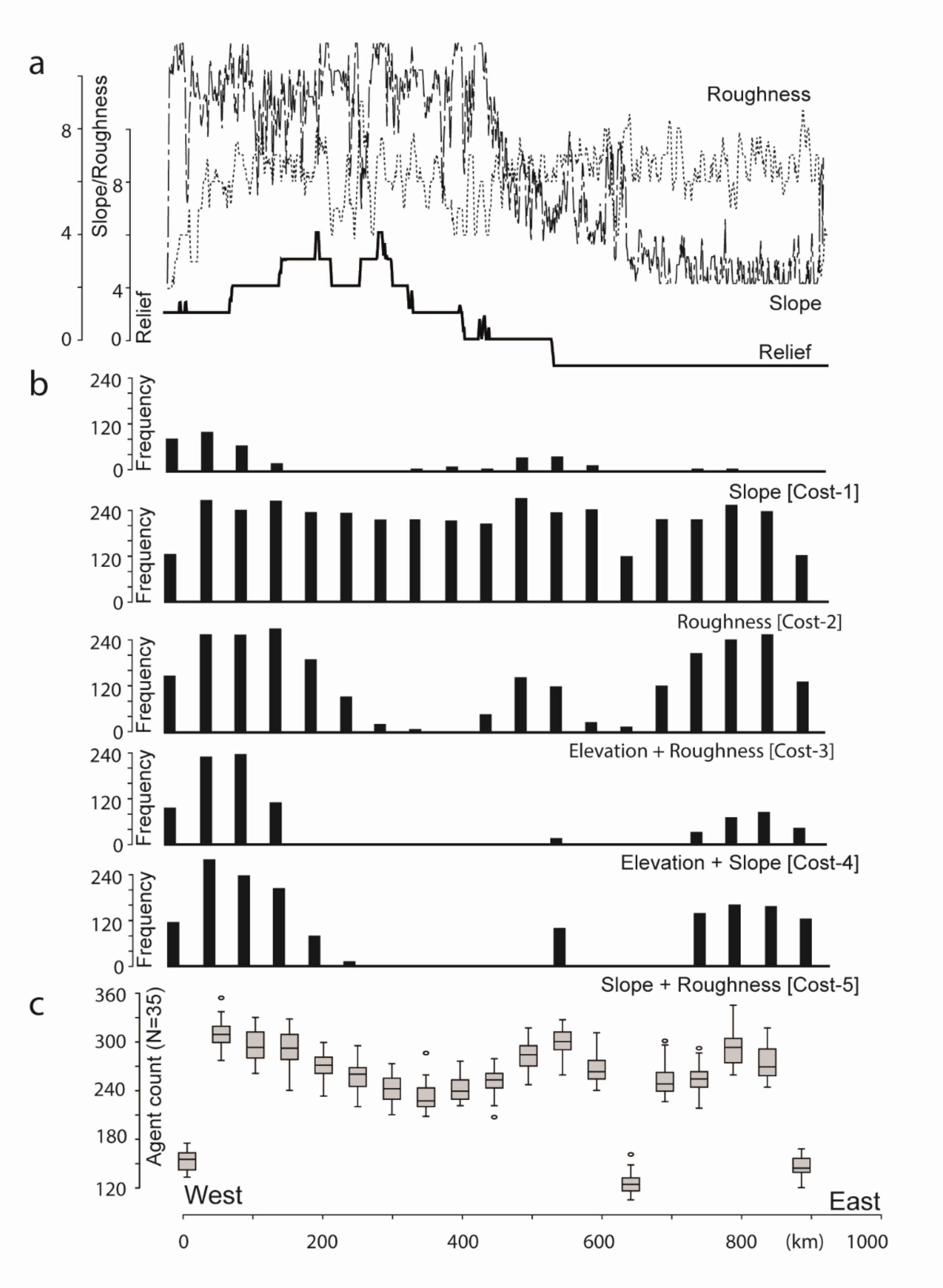


**Supplementary Figure 10:** Results of a sensitivity experiment conducted along a horizontal transect across the southern rift. Water-patches were placed at regular 10 km intervals along this transect and the agent number moving between patches output. Agent movements are the sum of those moving in either direction. **a**. Relief, slope and roughness data in ten equal classes along the transect. **b**. Frequency histograms of agent movements using various possible cost layers. Note that 'zero-cost' is not shown since agents 'leap-frog' stations along the transect. **c**. Replication experiment using roughness [Cost-2] as the cost layer. Note the absolute number of agents does vary but the relative proportion is always constant as is the network pattern. Roughness [Cost-2] was used as the default cost layer since it offered the least conservative option and therefore tests for possible population isolation.

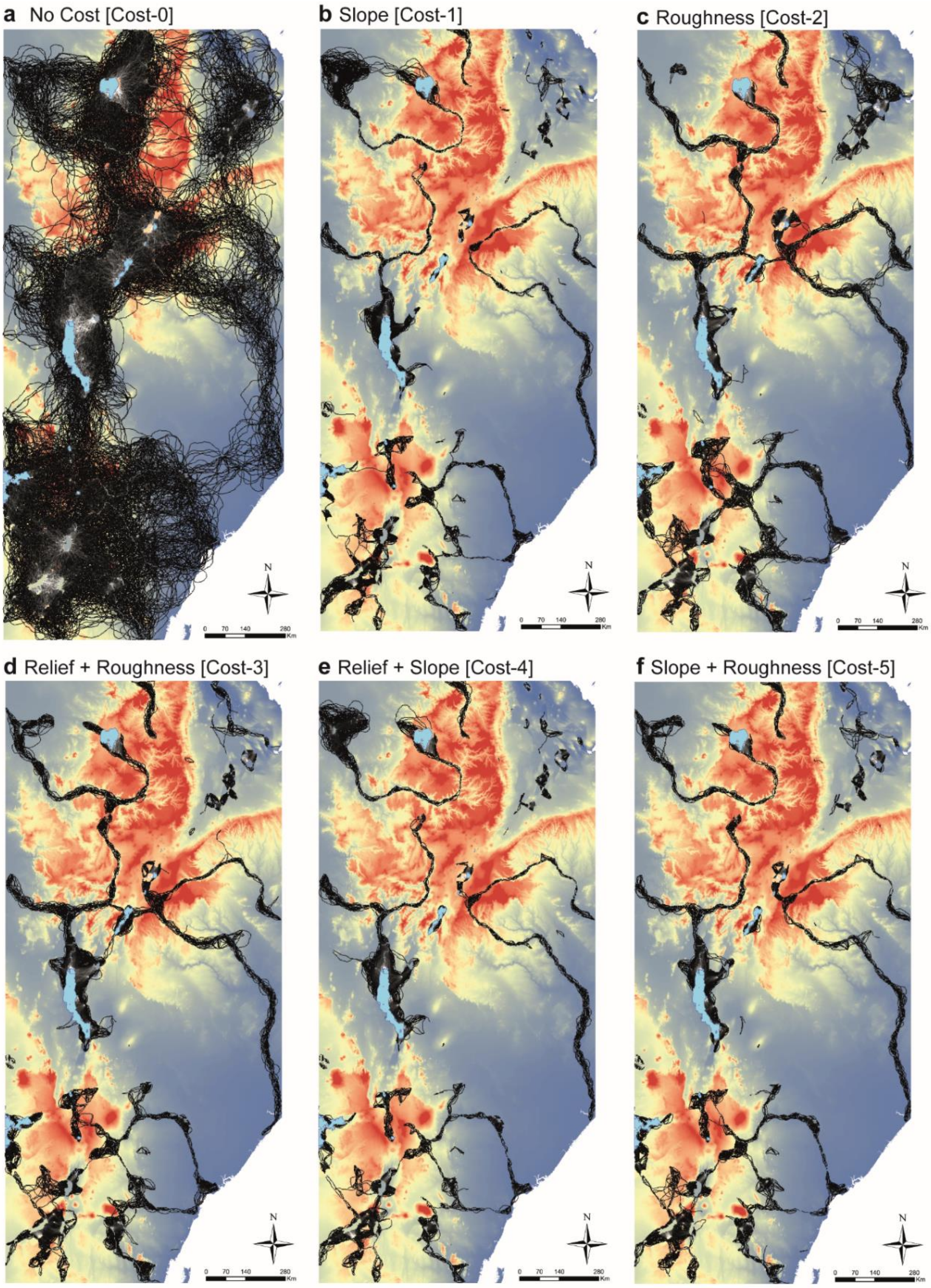


**Supplementary Figure 11:**
Runs to illustrate sensitivity to different cost layers. The hydrological system is based on the present day perennial springs, wetlands, lakes (saline + fresh) and major rivers with a flow rate greater than zero. In all cases a three day travel distance of 150 km, turn angle of 20 degrees and roughness as the cost layer with the standard [Scaling-2] was used. **a**. No-cost layer (Cost-0). **b**. Slope as the cost layer (Cost-1). **c**. Roughness as the cost layer (Cost-2). **d**. Relief and roughness as the cost layer (Cost-3). **e**. Relief and slope as the cost layer (Cost-4). f. Slope and roughness as the cost layer (Cost-5).

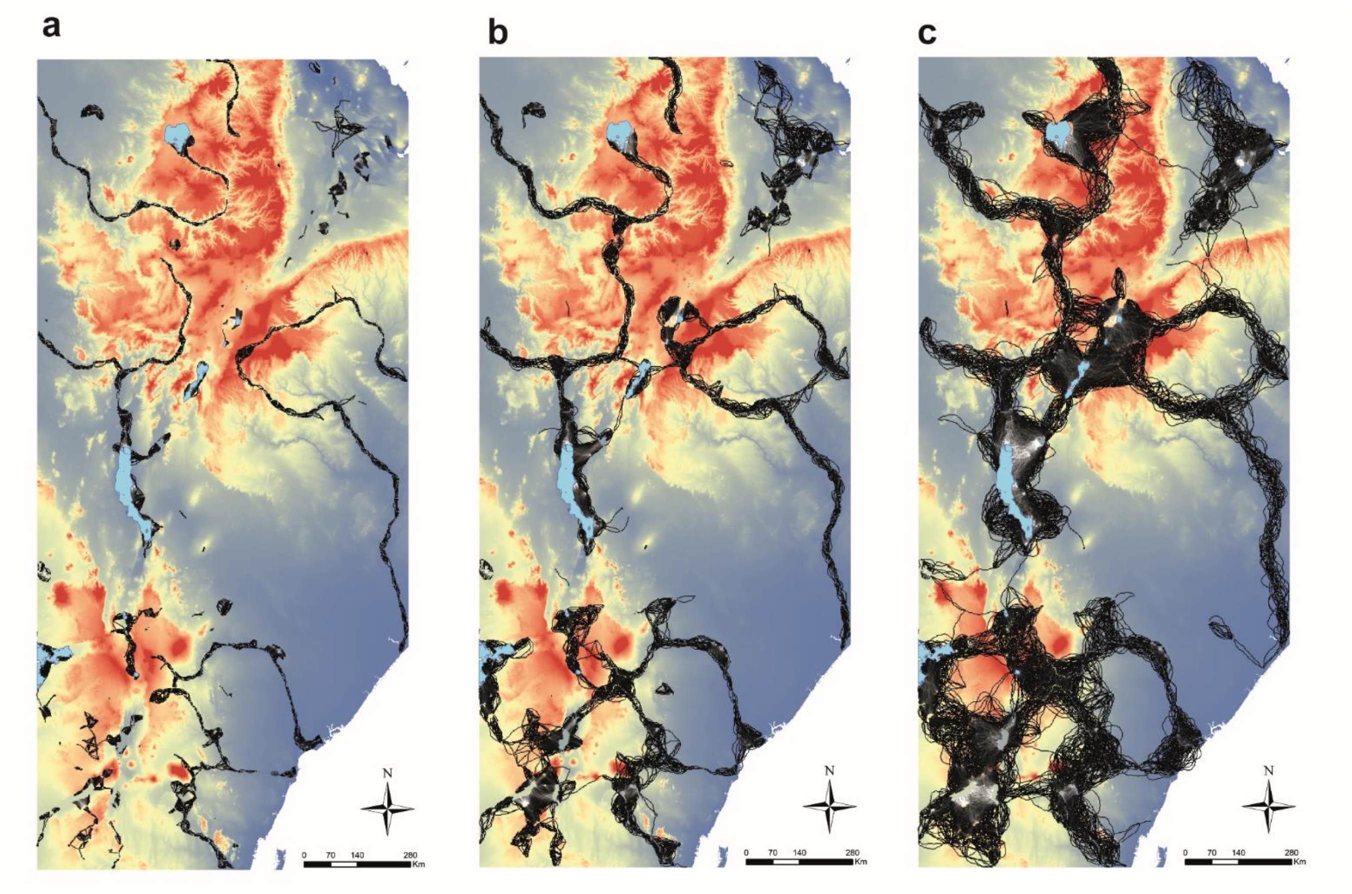


**Supplementary Figure 12:** Runs to illustrate sensitivity to different scaling of cost layers, in this case roughness [Cost-2]. The hydrological system is based on the present day perennial springs, wetlands, lakes (saline + fresh) and major rivers with a flow rate greater than zero. In all cases a three day travel distance of 150 km, turn angle of 20 degrees. **a**. Scenario with scaling 1. **b**. Scenario with scaling 2. **c**. Scenario with scaling 3.

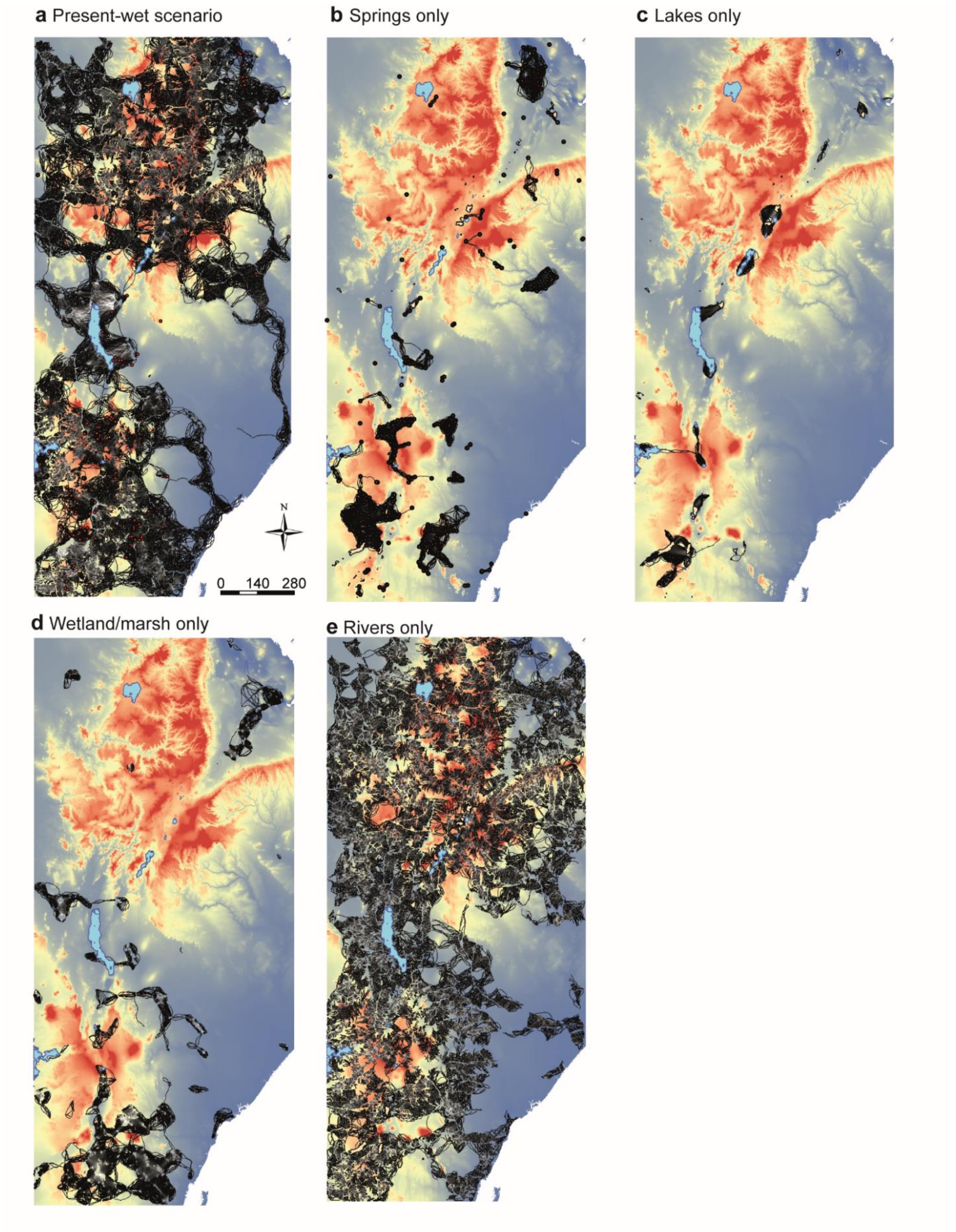


**Supplementary Figure 13:** Component parts of the hydrological system modelled with a three day travel distance of 150 km, turn angle of 20 degrees and using roughness as the cost layer with the standard [Scaling-2]. **a**. Present-day wet scenario. **b**. Springs only. **c**. Lakes only. d. Wetland/marsh only. **e**. Rivers only.

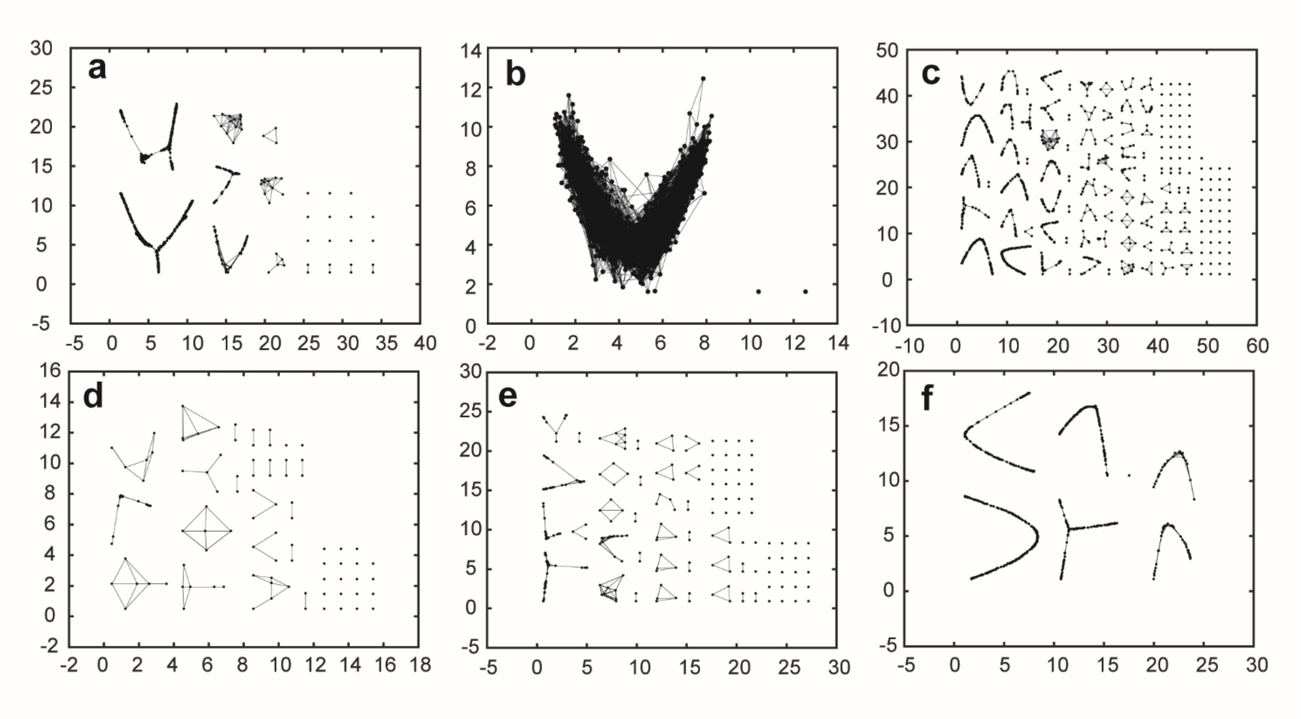


**Supplementary Figure 14: Sub-network shapes derived from successful journey matrices across various model runns.** **a.** Run-4 (Supplementary Table 3) in which a cost layer based on topographic roughness was used. Water elements correspond to the present day scenario. **b.** Run-9 (Supplementary Table 3) in which no cost layer was used. Water elements correspond to the present day scenario as in (a). **c.** Run-5 (Supplementary Table 3) in which a cost layer based on slope was used. Water elements correspond to the present day scenario as in (a). Note the increase in the number of sub-networks with use of slope as the coast layer compared to roughness (a). **d.** Run-15 (Supplementary Table 3) in which lakes were included as the only water source in the scenario. **e.** Run-14 (Supplementary Table 3) in which perennial springs were included as the only water source in the scenario. **f.** Run-14 (Supplementary Table 3) in which major were included as the only water source in the scenario.

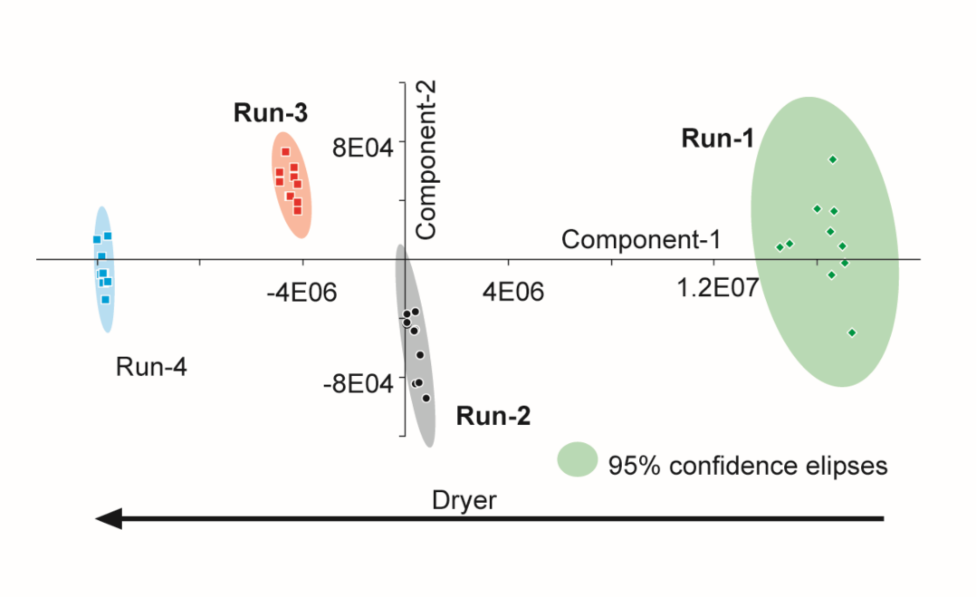


**Supplementary Figure 15:** Principle components analysis of the network and Fragstats connectivity metrics recorded for the four principle scenarios modelled based on a minimum of ten repetitions. The first component explains over 99.2% of the variance and gives good statistical separation as shown by the 95% confidence ellipses. The 95% confidence ellipses show the significant level of difference between the three primary runs modelled.

**Supplementary Table 1**: Coefficient of determination matrix calculated by applying linear regressions between all modelled spring variables. This indicates the primary control of the groundwater response time on the spring flow recession characteristics ($R^2$=0.23 to 0.64) as well as the lack of correlation between climatic (actual recharge) and spring recession timescales ($R^2$=0.01 to 0.02).

| | Time to recede to 1000 $m^3$/a | Time to recede to 90% of initial flow | Groundwater response time | Potential Recharge | Actual Recharge | Catchment Area | Hydraulic conductivity | Slope | Catchment length ($B_x$) | Specific yield |
|---|---|---|---|---|---|---|---|---|---|---|
| **Time to recede to 1000 $m^3$/a** | 1.00 | | | | | | | | | |
| **Time to recede to 90% of initial flow** | 0.25 | 1.00 | | | | | | | | |
| **Groundwater response time** | 0.23 | 0.64 | 1.00 | | | | | | | |
| **Potential Recharge** | 0.00 | 0.00 | 0.00 | 1.00 | | | | | | |
| **Actual Recharge** | 0.01 | 0.02 | 0.02 | 0.71 | 1.00 | | | | | |
| **Catchment Area** | 0.00 | 0.00 | 0.00 | 0.00 | 0.00 | 1.00 | | | | |
| **Hydraulic conductivity** | 0.01 | 0.01 | 0.01 | 0.01 | 0.00 | 0.01 | 1.00 | | | |
| **Slope** | 0.01 | 0.02 | 0.00 | 0.00 | 0.00 | 0.00 | 0.02 | 1.00 | | |
| **Catchment length ($B_x$)** | 0.00 | 0.00 | 0.01 | 0.00 | 0.02 | 0.03 | 0.00 | 0.01 | 1.00 | |
| **Specific yield** | 0.03 | 0.04 | 0.05 | 0.02 | 0.05 | 0.00 | 0.03 | 0.00 | 0.01 | 1.00 |

**Supplementary Table 2**: Typical network metrics and Fragstat output for selected model runs, showing the differences between the four modelled hydrological scenarios. Mean data is shown for a minimum of ten model repeats. See Supplementary Table 3 for the run definitions and Supplementary Table 4 for description of the metrics.

| | Network Numbers | Nodes | Edges | % Path by Area | NP - Total | NP-Paths | PD | PD [Path Class] | LPI-Path | TE [Path Class] | ED [Path Class] | LSI | AREA_AM | AREA_AM [Path Class] | PROX_AM | PROX_AM [Path Class] | CONNECT | CONNECT [Path Class] | COHESION | COHESION [Path Class] |
|---|---|---|---|---|---|---|---|---|---|---|---|---|---|---|---|---|---|---|---|---|
| Run_1 | Wet scenario; 150 km in three days; 20º turn angle; cost map-2 (roughness) with normal scaling; all perennial and seasonal water bodies. | | | | | | | | | | | | | | | | | | | |
| Min | 2 | 1664 | 10199 | 47.7971 | 4717 | 39 | 29249500 | 241833 | 45.67 | 2755.55 | 170868.2 | 55.19 | 0.0064 | 0.007 | 5182 | 2619.663 | 73.6737 | 74.0056 | 99.8803 | 99.9249 |
| Max | 6 | 1664 | 10557 | 49.0522 | 5168 | 59 | 32046090 | 365851 | 47.55 | 2846.94 | 176535.3 | 56.99 | 0.007 | 0.0074 | 9948 | 4832.677 | 74.4237 | 79.8335 | 99.8996 | 99.9339 |
| Mean | 3.7 | 1664 | 10305.4 | 48.1976 | 5002. | 48 | 31022330 | 297641 | 46.37 | 2807.20 | 174071.2 | 56.20 | 0.00675 | 0.00719 | 7278 | 3591.396 | 74.06493 | 76.58844 | 99.89101 | 99.9281 |
| Std. error | 0.495535 | 0 | 36.6082 | 0.12194 | 46.40 | 1.68 | 287735.6 | 10458 | 0.178 | 9.36999 | 581.0208 | 0.184 | 6.87184E-05 | 4.06885E-05 | 476 | 253.2112 | 0.08726276 | 0.6881662 | 0.001979251 | 0.000851535 |
| Stand. dev | 1.567021 | 0 | 115.7653 | 0.38563 | 146.7 | 5.33 | 909899.7 | 33071 | 0.563 | 29.6305 | 1837.349 | 0.583 | 0.000217307 | 0.000128668 | 1505 | 800.724 | 0.2759491 | 2.176173 | 0.00625894 | 0.002692789 |
| 95% +/- | 1.121 | | 82.5 | 0.2755 | 105 | 3.81 | 651000 | 23660 | 0.403 | 21.2 | 1315 | 0.417 | 0.0001555 | 9.2E-05 | 1076 | 572.8 | 0.197 | 1.5565 | 0.004 | 0.002 |
| N | 10 | 10 | 10 | 10 | 10 | 10 | 10 | 10 | 10 | 10 | 10 | 10 | 10 | 10 | 10 | 10 | 10 | 10 | 10 | 10 |
| Run_2 | Present-day wet scenario; 150 km in three days; 20º turn angle; cost map 2 (roughness) with normal scaling; permanent springs (+hydrothermal) major rivers, perennial rivers, saline lakes, perennial wetlands, and fresh lakes. | | | | | | | | | | | | | | | | | | | |
| Min | 11 | 935 | 5322 | 26.8003 | 2382 | 43 | 14770470 | 266637.4 | 14.0531 | 1721.088 | 106722.4 | 34.8385 | 0.0082 | 0.0014 | 3349.775 | 1607.645 | 70.0161 | 79.0909 | 99.8366 | 99.6255 |
| Max | 17 | 935 | 5523 | 27.4832 | 2504 | 53 | 15526980 | 328646.1 | 16.3565 | 1758.848 | 109063.9 | 35.5819 | 0.0085 | 0.0018 | 5680.048 | 4952.547 | 71.8227 | 89.258 | 99.8588 | 99.7128 |
| Mean | 13.6 | 935 | 5430.7 | 27.16322 | 2430 | 46.7 | 15068110 | 289580.6 | 15.04826 | 1739.594 | 107869.9 | 35.20564 | 0.00842 | 0.0016 | 4232.159 | 3605.783 | 70.96984 | 85.8662 | 99.84932 | 99.67257 |
| Std. error | 0.6 | 0 | 19.85898 | 0.0765512 | 12.50866 | 1.183685 | 77564.59 | 7339.878 | 0.2045157 | 4.520855 | 280.3323 | 0.08968132 | 2.90593E-05 | 3.65148E-05 | 239.7338 | 315.1601 | 0.1947095 | 0.9426458 | 0.002123823 | 0.008224112 |
| Stand. dev | 1.897367 | 0 | 62.79959 | 0.2420761 | 39.55587 | 3.743142 | 245280.8 | 23210.73 | 0.6467356 | 14.2962 | 886.4886 | 0.2835973 | 9.18937E-05 | 0.00011547 | 758.1047 | 996.6238 | 0.6157256 | 2.980908 | 0.006716117 | 0.02600692 |
| 95% +/- | 1.357 | | 44.9 | 0.173 | 28.3 | 2.678 | 175500 | 16600 | 0.4625 | 10.2 | 630 | 0.203 | 6.57E-05 | 8.26E-05 | 542.35 | 712.95 | 0.4405 | 2.1325 | 0.0045 | 0.0185 |
| N | 10 | 10 | 10 | 10 | 10 | 10 | 10 | 10 | 10 | 10 | 10 | 10 | 10 | 10 | 10 | 10 | 10 | 10 | 10 | 10 |

**Supplementary Table 2**: Continued.

| | Network Numbers | Nodes | Edges | % Path by Area | NP - Total | NP-Paths | PD | PD [Path Class] | LPI-Path | TE [Path Class] | ED [Path Class] | LSI | AREA _AM | AREA_AM [Path Class] | PROX _AM | PROX _AM [Path Class] | CONNECT | CONNECT [Path Class] | COHESION | COHESION [Path Class] |
|---|---|---|---|---|---|---|---|---|---|---|---|---|---|---|---|---|---|---|---|---|
| Run_3 | Present-day scenario; 150 km in three days; 20º turn angle; cost map 2 (roughness) with normal scaling; permanent springs (+hydrothermal) major rivers, saline lakes, perennial wetlands, and fresh lakes. | | | | | | | | | | | | | | | | | | | |
| Min | 36 | 1423 | 7360 | 10.6608 | 1590 | 22 | 9859382 | 136419.1 | 2.7947 | 895.36 | 55520.1 | 18.6754 | 0.0126 | 0.0003 | 1657.192 | 264.8213 | 69.5816 | 67.3333 | 99.8283 | 99.1715 |
| Max | 45 | 1423 | 7695 | 10.9226 | 1704 | 29 | 10566280 | 179825.2 | 4.7081 | 940.544 | 58321.9 | 19.5696 | 0.0126 | 0.0005 | 2135.501 | 2109.173 | 72.7804 | 78.6325 | 99.8442 | 99.3901 |
| Mean | 41.18182 | 1423 | 7497.091 | 10.79555 | 1656.727 | 25.81818 | 10273150 | 160095.2 | 4.383609 | 918.368 | 56946.8 | 19.12879 | 0.0126 | 0.000454546 | 1889.233 | 981.5265 | 71.31398 | 74.43701 | 99.83795 | 99.32158 |
| Std. error | 0.7724598 | | 29.11652 | 0.02554877 | 12.15139 | 0.6152336 | 75349.16 | 3814.983 | 0.1657019 | 4.426748 | 274.4969 | 0.08746044 | 0 | 2.07305E-05 | 57.57262 | 157.2008 | 0.3461581 | 0.9476671 | 0.001550036 | 0.01837311 |
| Stand. dev | 2.561959 | | 96.56858 | 0.0847357 | 40.30159 | 2.040499 | 249904.9 | 12652.87 | 0.549571 | 14.68186 | 910.4032 | 0.2900735 | 0 | 6.87552E-05 | 190.9468 | 521.3761 | 1.148076 | 3.143056 | 0.005140888 | 0.0609367 |
| 95% +/- | 1.721 | | 64.9 | 0.0565 | 27.05 | 1.371 | 168000 | 8505 | 0.3692 | 9.865 | 611.5 | 0.195 | 0 | 0.00004619 | 128.25 | 350.27 | 0.771 | 2.112 | 0.003 | 0.041 |
| N | 11 | 11 | 11 | 11 | 11 | 11 | 11 | 11 | 11 | 11 | 11 | 11 | 11 | 11 | 11 | 11 | 11 | 11 | 11 | 11 |
| Run_4 | Dry scenario; 150 km in three days; 20º turn angle; cost map 2 (roughness) with normal scaling; 23ka permanent springs low flow rivers and fresh lakes. | | | | | | | | | | | | | | | | | | | |
| Min | 34 | 563 | 2792 | 3.1442 | 440 | 26 | 2728382 | 161222.6 | 0.888 | 277.408 | 17201.71 | 6.5309 | 0.015 | 0.0001 | 395.3072 | 3.6466 | 78.3896 | 69.8851 | 99.8544 | 98.0315 |
| Max | 45 | 563 | 2947 | 3.2999 | 510 | 33 | 3162443 | 204628.7 | 1.0574 | 301.408 | 18689.92 | 7.0031 | 0.0151 | 0.0001 | 545.2174 | 61.0123 | 82.5537 | 77.5132 | 99.8648 | 98.2058 |
| Mean | 39.6 | 563 | 2868.8 | 3.24589 | 482 | 29.6 | 2988819 | 183545.7 | 0.98136 | 293.6032 | 18205.95 | 6.84925 | 0.01502 | 0.0001 | 475.5652 | 33.77266 | 80.01442 | 74.11673 | 99.85791 | 98.1179 |
| Std. error | 1.0873 | 0 | 15.28456 | 0.01603512 | 6.492731 | 0.718022 | 40260.58 | 4452.36 | 0.01909467 | 2.407098 | 149.261 | 0.04737585 | 1.33333E-05 | 4.51751E-21 | 13.96711 | 7.591681 | 0.3902411 | 0.900199 | 0.000966489 | 0.01950178 |
| Stand. dev | 3.438346 | 0 | 48.33402 | 0.05070752 | 20.53182 | 2.270585 | 127315.1 | 14079.6 | 0.06038264 | 7.611912 | 472.0047 | 0.1498156 | 4.21637E-05 | 1.42856E-20 | 44.16789 | 24.007 | 1.234051 | 2.846679 | 0.003056305 | 0.06167005 |
| 95% +/- | 2.46 | 0 | 34.6 | 0.0363 | 14.69 | 1.624 | 91100 | -92000 | 0.043215 | 5.445 | 338 | 0.10715 | 3E-05 | 0 | 31.595 | 17.1735 | 0.8825 | 2.0365 | 0.002 | 0.044 |
| N | 10 | 10 | 10 | 10 | 10 | 10 | 10 | 10 | 10 | 10 | 10 | 10 | 10 | 10 | 10 | 10 | 10 | 10 | 10 | 10 |

**Supplementary Table 3**: Main model runs used, bold text highlights the significant difference between each run.

| | |
|---|---|
| Run_1 | **Wet scenario; 150 km in three days; 20º turn angle; cost map-2 (roughness) with normal scaling; all perennial and seasonal water bodies.** |
| Run_2 | **Present-day wet scenario; 150 km in three days; 20º turn angle; cost map 2 (roughness) with normal scaling; permanent springs (+hydrothermal) major rivers, perennial rivers, saline lakes, perennial wetlands, and fresh lakes.** |
| Run_3 | **Present-day scenario; 150 km in three days; 20º turn angle; cost map 2 (roughness) with normal scaling; permanent springs (+hydrothermal) major rivers, saline lakes, perennial wetlands, and fresh lakes.** |
| Run_4 | **Dry scenario; 150 km in three days; 20º turn angle; cost map 2 (roughness) with normal scaling; 23ka permanent springs low flow rivers and fresh lakes.** |
| Run_5 | Dry scenario; 150 km in three days; 20º turn angle; cost map-2 roughness with normal scaling; 23ka permanent springs **(+hydrothermal)** low flow rivers and fresh lakes. |
| Run_6 | Present-day scenario; 150 km in three days; 20º turn angle; **cost map 1 (slope)** with normal scaling; permanent springs (+hydrothermal) major rivers, saline lakes, perennial wetlands, and fresh lakes. |
| Run_7 | Present-day scenario; 150 km in three days; 20º turn angle; **cost map 3 (relief + roughness**) with normal scaling; permanent springs (+hydrothermal) major rivers, saline lakes, perennial wetlands, and fresh lakes. |
| Run_8 | Present-day scenario; 150 km in three days; 20º turn angle; **cost map 4 (relief+ slope)** with normal scaling; permanent springs (+hydrothermal) major rivers, saline lakes, perennial wetlands, and fresh lakes. |
| Run-9 | Present-day scenario; 150 km in three days; 20º turn angle; **cost map 5 (slope + roughness)** with normal scaling; permanent springs (+hydrothermal) major rivers, saline lakes, perennial wetlands, and fresh lakes. |
| Run_10 | Present-day scenario; 150 km in three days; 20º turn angle; **cost map zero (no cost)** with normal scaling; permanent springs (+hydrothermal) major rivers, saline lakes, perennial wetlands, and fresh lakes. |
| Run_11 | Present-day scenario; **180 km in three days**; 20º turn angle; cost map 2 (roughness) with normal scaling; permanent springs (+hydrothermal) major rivers, saline lakes, perennial wetlands, and fresh lakes. |
| Run_12 | Present-day scenario; **120 km in three days**; 20º turn angle; cost map 2 (roughness) with normal scaling; permanent springs (+hydrothermal) major rivers, saline lakes, perennial wetlands, and fresh lakes. |
| Run_13 | Present-day scenario; 150 km in three days; 20º turn angle; cost map 2 (roughness) with **low scaling;** permanent springs (+hydrothermal) major rivers, saline lakes, perennial wetlands, and fresh lakes. |
| Run_14 | Present-day scenario; 150 km in three days; 20º turn angle; cost map 2 (roughness) with **very low scaling**; permanent springs (+hydrothermal) major rivers, saline lakes, perennial wetlands, and fresh lakes. |
| Run_15 | Present-day scenario; 150 km in three days; 20º turn angle; cost map 2 (roughness) with normal scaling; **permanent springs (+hydrothermal) only**. |
| Run_16 | Present-day scenario; 150 km in three days; 20º turn angle; cost map 2 (roughness) with normal scaling; **saline lakes and fresh lakes only.** |
| Run_17 | Present-day scenario; 150 km in three days; 20º turn angle; cost map 2 (roughness) with normal scaling; **major rivers only.** |

**Supplementary Table 4**: Metric definitions.

| Metrics | Description |
|---|---|
| Network Types | Classification of sub-network types [Matlab output] |
| Network Numbers | Total number of sub-networks [Matlab output] |
| Nodes | Number of nodes [Matlab output] |
| Edges | Number of edges between nodes [Matlab output] |
| % PATH by Area | Percentage landcover patch area containing successful routes [PATH; Fragstats output] |
| NP -Total | Total number of landcover patches [PATH and Non-PATH; Fragstats output] |
| NP-PATH | Total number of landcover patches [PATH only; Fragstats output] |
| PD | Total patch density all types [PATH and Non-PATH; Fragstats output] |
| PD [PATH Class] | Total patch density of patches contains successful routes [PATH only; Fragstats output] |
| LPI [PATH Class] | Larges patch index for patches containing successful routes [PATH only; Fragstats output] |
| TE [PATH Class] | Total edge length of patches containing successful routes [PATH only; Fragstats output] |
| ED [PATH Class] | Total edge density of patches containing successful routes [PATH only; Fragstats output] |
| LSI | Landscape shape index [PATH and Non-PATH; Fragstats output] |
| AREA_AM | Area-weighted mean patch size [PATH and Non-PATH; Fragstats output] |
| AREA_AM [PATH Class] | Area-weighted mean patch size [PATH only; Fragstats output] |
| PROX_AM [PATH Class] | Area-weighted mean proximity index [PATH only; Fragstats output] |
| CONNECT | Patch connectance index [PATH and Non-PATH; Fragstats output] |
| CONNECT [Path Class] | Patch connectance index [PATH only; Fragstats output] |
| COHESION | Patch cohesion index [PATH and Non-PATH; Fragstats output] |
| COHESION [Path Class] | Patch cohesion index [PATH only; Fragstats output] |

**Supplementary Table 5**: Typical network metrics and Fragstat output for selected model runs, showing the impact of different costing and scaling scenarios. See Supplementary Table 3 for the run definitions and Supplementary Table 4 for description of the metrics.

| Run ID | Network # | Nodes | Edges | % Path by Area | NP - Total | NP-Paths | PD | PD [Path Class] | LPI-Path | TE [Path Class] | ED [Path Class] | LSI | AREA_AM | AREA_AM [Path Class] | PROX_AM | PROX_AM [Path Class] | CONNECT | CONNECT [Path Class] | COHESION | COHESION [Path Class] |
|---|---|---|---|---|---|---|---|---|---|---|---|---|---|---|---|---|---|---|---|---|
| Run_5 | 40 | 563 | 2792 | 3.1836 | 451 | 26 | 2796592 | 161223 | 0.9315 | 306.496 | 19005 | 7.1033 | 0.0151 | 0.0001 | 490.524 | 34.7232 | 81.3094 | 80.9231 | 99.8604 | 98.0503 |
| Run_6 | 151 | 1423 | 4725 | 3.3536 | 569 | 262 | 3528295 | 1624628 | 0.5343 | 350.784 | 21751 | 7.9707 | 0.015 | 0 | 310.2586 | 17.1406 | 70.7462 | 73.8323 | 99.8671 | 95.1152 |
| Run_7 | 116 | 1423 | 5726 | 4.2208 | 543 | 120 | 3367072 | 744104 | 0.497 | 417.312 | 25876 | 9.2771 | 0.0147 | 0 | 483.8752 | 73.9674 | 75.4266 | 70.7423 | 99.8625 | 96.8689 |
| Run_8 | 145 | 1423 | 4905 | 3.6225 | 548 | 208 | 3398076 | 1289781 | 0.6143 | 360.032 | 22325.1137 | 8.1524 | 0.0149 | 0 | 310.057 | 43.0555 | 70.6599 | 70.5732 | 99.8659 | 95.9574 |
| Run-9 | 120 | 1423 | 5363 | 3.4614 | 446 | 167 | 2765588 | 1035545 | 0.3105 | 366.912 | 22751.7335 | 8.2875 | 0.015 | 0 | 274.0212 | 57.3465 | 74.7198 | 69.7208 | 99.8728 | 94.9773 |
| Run_10 | 3 | 1423 | 9631 | 87.0912 | 10212 | 4 | 6.3E+07 | 24803.5 | 87.0887 | 1813.73 | 112466.9022 | 36.449 | 0.0123 | 0.014 | 125.3557 | 7.9998 | 68.3978 | 100 | 99.8687 | 99.9909 |
| Run_11 | 80 | 1423 | 6514 | 5.7147 | 703 | 82 | 4359211 | 508471 | 1.1163 | 522.72 | 32413.1839 | 11.3454 | 0.0142 | 0.0001 | 832.146 | 237.7534 | 76.4149 | 67.9012 | 99.8546 | 98.1108 |
| Run_12 | 126 | 1423 | 5490 | 3.4211 | 462 | 153 | 2864802 | 948733 | 0.1776 | 368.8 | 22868.8059 | 8.3246 | 0.015 | 0 | 332.7373 | 63.669 | 74.2088 | 69.0488 | 99.8688 | 94.8065 |
| Run_13 | 36 | 1423 | 7559 | 10.8873 | 1693 | 22 | 1E+07 | 136419 | 4.6923 | 931.2 | 57742.4947 | 19.3785 | 0.0126 | 0.0005 | 1886.1834 | 710.0526 | 72.8022 | 74.8918 | 99.8413 | 99.3703 |
| Run_14 | 15 | 1423 | 9703 | 24.3934 | 4454 | 8 | 2.8E+07 | 49607 | 24.265 | 1751.23 | 108591.607 | 35.5047 | 0.0073 | 0.0039 | 8385.3464 | 282.2207 | 74.3005 | 78.5714 | 99.822 | 99.8665 |
| Run_15 | 80 | 356 | 2094 | 1.5587 | 192 | 38 | 1190567 | 235633 | 0.5227 | 85.024 | 5272.2271 | 2.745 | 0.0156 | 0 | 225.3965 | 12.2607 | 68.2153 | 74.3954 | 99.8943 | 97.31 |
| Run_16 | 42 | 107 | 210 | 0.3848 | 46 | 29 | 285240 | 179825 | 0.0473 | 36.48 | 2262.0771 | 1.7897 | 0.016 | 0 | 10.392 | 4.7789 | 68.6347 | 67.734 | 99.9471 | 91.4097 |
| Run_17 | 7 | 865 | 5249 | 2.5892 | 286 | 14 | 1773449 | 86812.2 | 0.7697 | 228.96 | 14197.5103 | 5.5749 | 0.0153 | 0.0001 | 270.1602 | 72.5317 | 83.5359 | 71.4286 | 99.8803 | 98.141 |

**Supplementary Table 6**: Typical network metrics and Fragstat output for selected model runs, showing the impact of different costing and scaling scenarios. All results are statistically different at 95% except for those indicated with *.

| Metrics | Run_1 [N=10] | | Run_2 [N=10] | | Run_3 [N=11] | | Run_4 [N=10] | |
|---|---|---|---|---|---|---|---|---|
| | Mean | 95% +/- | Mean | 95% +/- | Mean | 95% +/- | Mean | 95% +/- |
| Network Numbers | 3.7 | 1.121 | 13.6 | 1.357 | 41.18182* | 1.721* | 39.6* | 2.46* |
| Edges | 10305.4 | 82.5 | 5430.7 | 44.9 | 7497.091 | 64.9 | 2868.8 | 34.6 |
| % Path by Area | 48.19762 | 0.2755 | 27.16322 | 0.173 | 10.79555 | 0.0565 | 3.24589 | 0.0363 |
| NP -Total | 5002.9 | 105 | 2430 | 28.3 | 1656.727 | 27.05 | 482 | 14.69 |
| PD | 3100000 | 651000 | 1510000 | 175500 | 10273150 | 168000 | 2988819 | 91100 |
| LPI-Path | 46.37813 | 0.403 | 15.04826 | 0.4625 | 4.383609 | 0.3692 | 0.98136 | 0.043215 |
| TE [Path Class] | 2807.206 | 21.2 | 1739.594 | 10.2 | 918.368 | 9.865 | 293.6032 | 5.445 |
| ED [Path Class] | 174071.2 | 1315 | 107869.9 | 630 | 56946.8 | 611.5 | 18205.95 | 338 |
| LSI | 56.20748 | 0.4175 | 35.20564 | 0.203 | 19.12879 | 0.195 | 6.84925 | 0.10715 |
| AREA_AM | 0.00675 | 0.0001555 | 0.00842 | 6.57E-05 | 0.0126 | 0 | 0.01502 | 3E-05 |
| PROX_AM | 7278.063 | 1076.95 | 4232.159 | 542.35 | 1889.233 | 128.25 | 475.5652 | 31.595 |
| PROX_AM [Path Class] | 3591.396 | 572.8 | 3605.783 | 712.95 | 981.5265 | 350.27 | 33.77266 | 17.1735 |
| CONNECT [Path Class] | 76.58844 | 1.5565 | 85.8662 | 2.1325 | 74.43701 | 2.112 | 74.11673 | 2.0365 |
| COHESION [Path Class] | 99.9281 | 0.002 | 99.67257 | 0.0185 | 99.32158 | 0.041 | 98.1179 | 0.044 |
